# Identification of Novel Laminin- and Fibronectin-binding Proteins by Far-Western Blot: Capturing the Adhesins of *Streptococcus suis* Type 2

**DOI:** 10.3389/fcimb.2015.00082

**Published:** 2015-11-16

**Authors:** Quan Li, Hanze Liu, Dechao Du, Yanfei Yu, Caifeng Ma, Fangfang Jiao, Huochun Yao, Chengping Lu, Wei Zhang

**Affiliations:** Key Lab of Animal Bacteriology, OIE Reference Lab for Swine Streptococcosis, College of Veterinary Medicine, Ministry of Agriculture, Nanjing Agricultural UniversityNanjing, China

**Keywords:** *Streptococcus suis* serotype 2, interactions, surface proteins, LN- binding proteins, FN-binding proteins

## Abstract

Bacterial cell wall (CW) and extracellular (EC) proteins are often involved in interactions with extracellular matrix (ECM) proteins such as laminin (LN) and fibronectin (FN), which play important roles in adhesion and invasion. In this study, an efficient method combining proteomic analysis and Far-Western blotting assays was developed to screen directly for bacterial surface proteins with LN- and FN-binding capacity. With this approach, fifteen potential LN-binding proteins and five potential FN-binding proteins were identified from *Streptococcus suis* serotype 2 (SS2) CW and EC proteins. Nine newly identified proteins, including oligopeptide-binding protein OppA precursor (OppA), elongation factor Tu (EF-Tu), enolase, lactate dehydrogenase (LDH), fructose-bisphosphate aldolase (FBA), 3-ketoacyl-ACP reductase (KAR), Gly ceraldehyde-3-phosphate dehydrogenase (GAPDH), Inosine 5′-monophosphate dehydrogenase (IMPDH), and amino acid ABC transporter permease (ABC) were cloned, expressed, purified and further confirmed by Far-Western blotting and ELISA. Five proteins (OppA, EF-Tu, enolase, LDH, and FBA) exhibited specifically binding activity to both human LN and human FN. Furthermore, seven important recombinant proteins were selected and identified to have the ability to bind Hep-2 cells by the indirect immunofluorescent assay. In addition, four recombinant proteins, and their corresponding polyclonal antibodies, were observed to decrease SS2 adhesion to Hep-2 cells, which indicates that these proteins contribute to the adherence of SS2 to host cell surface. Collectively, these results show that the approach described here represents a useful tool for investigating the host-pathogen interactions.

## Introduction

*Streptococcus suis* (SS) is an important pathogen in swine and human that causes septicemia, meningitis, arthritis, and pneumonia (Gottschalk and Segura, 2000). It is also a potential threat of significance to public health, for humans can be infected with the bacteria through skin wounds or the consumption of raw pork (Kay et al., 1995; Yu et al., 2005; Gottschalk et al., 2007). Among the 33 serotypes, serotype 2 is the most commonly isolated and the most closely associated with the diseases (Gottschalk and Segura, 2000; Higgins and Gottschalk, 2000). So far, the virulence factors of *Streptococcus suis* serotype 2 (SS2) remain to be fully clarified, and the putative virulence factors, such as suilysin (SLY), extracellular protein factor (EF), and muramidase-released protein (MRP), cannot explain the pathogenesis of this bacterium (Staats et al., 1997; Berthelot-Hérault et al., 2005).

Extracellular matrix (ECM) is a composite of the secreted products of resident cells in every tissue and organ (Westerlund and Korhonen, 1993). During adhesion, ECM proteins, such as laminin (LN) and fibronectin (FN), usually serve as mediators between host cells and bacteria (Beachey and Courtney, 1987). Bacteria recognize ECM via their own ECM-binding proteins so as to adhere to endothelial and epithelial cells in host tissue. The ECM-binding proteins of bacteria usually make great contributions to the infection and host-pathogen interactions. A number of LN- and FN-binding proteins have shown to be important virulence factors of SS, and many SS proteins have been proposed to contribute to the colonization of organs because of their ability to bind LN and FN, such as FBPS (de Greeff et al., 2002), enolase (Esgleas et al., 2008; Zhang et al., 2009a), Ssa (Li et al., 2013), Lmb (Zhang et al., 2014b), and Autolysin (Ju et al., 2012). Therefore, large scale identification of the LN- and FN-binding proteins of SS2 which interact with host cells may provide a global view of the pathogenesis of SS2-induced infection.

Gram-positive pathogenic bacteria express specific surface-related proteins, including cell wall (CW) and extracellular (EC) proteins, that can interact with the factors of the host ECM (Gilbert et al., 1997; Beg et al., 2002; Kreikemeyer et al., 2004), adhere to host cells (Lee and Boran, 2003; Samen et al., 2007), invade, and evade host defenses (Dave et al., 2004; Peppoloni et al., 2006; Campos et al., 2008; Cron et al., 2009). Several CW-associated or EC proteins are significantly important for the pathogenesis of the SS infection (Fittipaldi et al., 2012). As for SS2, many CW and EC proteins have been identified as adhesion molecules and proposed to be virulence factors, including FBPS (de Greeff et al., 2002), GAPDH (Brassard et al., 2004), glutamate dehydrogenase (GDH) (Okwumabua et al., 2001), enolase (Esgleas et al., 2008; Zhang et al., 2009a), amylopullulanase (Ferrando et al., 2010), 6-phosphogluconate-dehydrogenase (6-PGD) (Tan et al., 2008), as well as a glutamine synthetase (Si et al., 2009).

Far-Western blot analysis is a method to identify protein-protein interactions that the unknown target protein is probed with a known antibody-detectable bait protein on the membrane. As described, Far-Western blot can be used to screen specific interacting proteins in a complex mixture of proteins. In this work, proteomic analysis together with Far-Western blotting assays identified 15 potential LN-binding proteins and five potential FN-binding proteins in SS2 surface proteins. All 15 potential LN-binding proteins and two potential FN-binding proteins were detected for the first time. Nine proteins were selected from the identified prey for further confirmation by Far-Western blotting and ELISA, and the adhesion of seven important proteins was confirmed by indirect immunofluorescence assays. Additionally, four recombinant proteins, and their corresponding polyclonal antibodies, were observed to clearly inhibit SS2 adhesion to Hep-2 cells, indicating that these proteins contribute to the adherence of SS2 to host cells. Taken together, these results show that the approach described here provides a useful clue to investigating the host-pathogen interactions.

## Materials and methods

### Bacterial strains and culture conditions

The SS2 strain ZY05719 was isolated from a diseased pig during an outbreak in Ziyang, China. The bacteria were cultured in Todd Hewitt Broth (THB; BD Inc.) or agar medium at 37°C and harvested at the late exponential phase of growth. A total of 50 μg/ml ampicillin (Amp; Sigma) and 50 μg/ml kanamycin (Kan; Sigma) was used for the *E. coli* transformants. *E. coli* strain DH5α and BL21 (DE3) was grown on Luria-Bertani (LB) agar plates or in LB broth. The pMD18-T vectors (Takara), pET-32a (+) and pET-28a (+) were used for protein expression, respectively.

### Preparation of CW and EC proteins

CW proteins were prepared as described previously with minor modifications (Ling et al., 2004; Wu et al., 2008). Bacterial cultures were separated by centrifugation at 4°C for 10 min at 12,000 × g. SS2 pellets were washed twice with PBS, then resuspended in 4 ml sample preparation solution [30 mM Tris-HCl (pH 7.5), 25% sucrose, 125 U/ml mutanolysin (Sigma), 3 mM MgCl_2_], and incubated at 37°C for 1.5 h. The cell lysate was centrifuged at 4°C for 10 min at 12,000 × g. Proteins in the supernatant were precipitated in 10% pre-chilled (4°C) trichloroacetic acid (TCA, Sinopharm Chuan Kang Pharmaceutical Co., Ltd. China) and incubated in ice-water for 0.5 h, then centrifuged at 4°C for 10 min at 12,000 × g. The residual TCA was removed by washing the pellet twice using 10 ml chilled acetone, and then dried the pellet in air.

EC proteins were prepared as described previously (Zhang and Lu, 2007), Briefly, culture supernatant was centrifuged at 4°C for 10 min at 12,000 × g, and filtered twice through a 0.22 μm membrane filters in order to remove residual debris. Then, TCA was added to the supernatant at a concentration of 10% and the proteins were precipitated in ice-water. The residual TCA was removed by washing the pellet twice using 10 ml chilled acetone after centrifuged at 4°C for 10 min at 12,000 × g, and then air-dried.

### Identification of novel laminin- and fibronectin-binding proteins by 2D-far-western blot

The 2-DE experiment was carried out as previously described (Zhang and Lu, 2007). Briefly, protein samples (200 μg) were resuspended in 250 μl sample preparation solution and solubilized at 25°C for 30 min, then centrifuged at 25°C for 20 min at 13,000 × g. Two equivalent protein samples were resuspended in 250 μL rehydration solution (0.002% w/v bromophenol blue, 0.5% v/v IPG buffer, 0.2% w/v DTT, 2% w/v CHAPS, 2 M thiourea, 7 M urea) after treatment with 2-D Clean-up Kit (GE Healthcare). And then immobilized pH gradient (IPG) strips (Immobiline DryStrip, 13 cm and pH 4–7; GE Healthcare) were rehydrated at room temperature for 12 h. Isoelectric focusing (IEF) of the soluble samples was executed for 11.5 h at 20°C (maximun voltage: 8000 V, maximum current: 50 μA/IPG strip, total 28,000 V/h), which was performed in six steps as follows: 500 V, 4 h; 1000 V, 1 h; 2000 V, 1 h; 4000 V, 1 h; 8000 V, 2.5 h; and 8000 V for 2 h.

For Far-Western blot analysis, the CW and EC proteins were separated by 2-DE gels, and then transferred onto polyvinylidene difluoride (PVDF) membranes. The PVDF membranes were blocked with 5% (w/v) skimmed milk overnight at 4°C, diluted with TBST [150 mM NaCl, 50 mM Tris-HCl buffer (pH 7.4), 0.05% Tween 20]. Then, the membranes were washed and incubated with human LN (Sigma; 5 μg/ml) or human plasma FN (Sigma; 10 μg/ml) dissolved in 5% (w/v) skimmed milk for 24 h at 4°C. Meanwhile, the negative control was blocked with 1% BSA for 24 h at 4°C, followed by three times of wash with TBST. The membranes were incubated with rabbit anti-LN (Abcam; 1:2000 dilution) or rabbit anti-FN (Boster; 1:2000 dilution) antibody at 37°C for 2 h, rinsed three times with TBST and then incubated with goat anti-rabbit IgG antibody (Boster; 1:2000 dilution) at 37°C for 1 h. After three-time washing, the positive protein spots were developed using substrate solution 3,3′-diaminobenzidine (DAB; Tiangen, China).

### MALDI-TOF-MS and database searching

After comparison with the profiles of Western blotting by using ImageMaster 7.0, the spots were excised from the 2D gels for in-gel tryptic digestion (Nanjing Steed Biotechnologies Co. Ltd.) followed by MALDI-TOF-MS analysis. Peptide mass fingerprinting (PMF) data was performed with the MASCOT Software (http://www.matrixscience.com). The MASCOT searches were used to identify peptides which were considered significant and used to generate the combined peptide score. The criteria used to validate protein identifications were based on PMF data, including the number of matched peptides, the extent of sequence coverage, and the score probability. For accurate identification of protein spots, more than 5 peptides and 15% sequence coverage must be matched for MALDI-TOF data. The proteins with lower Mascot score were either rejected or verified manually.

### Cloning, expression and purification of recombinant potential LN- and FN-binding proteins

To express and purify the recombinant LN- and FN-binding proteins, *S. suis* strain ZY05719 genomic DNA was used as template for PCR using the primers listed in Table 1, with PrimeSTAR HS DNA polymerase (Vazyme). A total of nine PCR products were inserted into the pET32a or pET28a vectors using the *Xho*I and *BamH* I sites and transformed into *E. coli* BL21 (DE3) for expression at 37°C. The cloned sequences were confirmed by DNA sequence analysis. Recombinant proteins were induced with 1 mM IPTG at 37°C for 5 h when the cell had grown to an OD600 of 0.6. Bacterial cultures were harvested by centrifugation at 4°C for 10 min at 12,000 × g. The induced proteins were purified from cultures by Ni-chelating chromatography (GE Healthcare) according to the manufacturer's recommendations.

**Table 1:** **Primers used in this study**.

**Primers**	**Sequences (5′–3′)^a^**	**Length of PCR products (bp)**	**Expression vector**
OppA-F	CGCGGATCCTTGACAGGTATGCAAGAATTT	1311	pET-32a
OppA-R	CCGCTCGAGTTATTCTGCCACTACACCCTT		
EF-Tu-F	CGCGGATCCTTGGAGGCTTTTACCAAAATG	1215	pET-32a
EF-Tu-R	CCGCTCGAGTTAAGCTTCGATTTCTGTAAC		
enolase-F	CGCGGATCCATGTCAATTATTACTGATGTT	1308	pET-32a
enolase-R	CCGCTCGAGTTATTTTTTCAAGTTGTAGAA		
LDH-F	CGCGGATCCTCTGCTTATGCTTATGCTCTT	843	pET-28a
LDH-R	CCGCTCGAGTTTTTGCAATTCAGCTTCGCT		
FBA-F	CGCGGATCCACAAACAACCTTGAGTGGACT	798	pET-32a
FBA-R	CCGCTCGAGCGCTGAACCGAATACGTCGAT		
KAR-F	CGCGGATCCCTGCATGGTCAAGCCTCCGTC	732	pET-32a
KAR-R	CCGCTCGAGGTAACAGGTTCAAGTCGAGGA		
GAPDH-F	CGCGGATCCGAACGTATCGGTCGTCTTGCT	972	pET-32a
GAPDH-R	CCGCTCGAGAATTTTTGCGAAGTACTCAAG		
IMPDH-F	CGCGGATCCGTTTGGAGCTTCGTTTGTGAT	1461	pET-32a
IMPDH-R	CCGCTCGAGAACTGGGACACTAAATTTTTG		
ABC-F	CGCGGATCCATCATCAACGAAGTAGCCAAG	1296	pET-32a
ABC-R	CCGCTCGAGCCTGAGTTCAACTAGGGCAGC		
MRP-F	CGCGGATCCCAGAAAGTTGCAAACTGGATT	1104	pET-32a
MRP-R	CCGCTCGAGACCAGTATTTGGCAATTGAGC		

a *The underlined sequences are the restriction sites*.

### Polyclonal antibody production of recombinant potential LN- and FN-binding proteins

Polyclonal antibody against the purified recombinant proteins (OppA, EF-Tu, and enolase, LDH, FBA, IMPDH, and ABC) was prepared by immunizing rabbits subcutaneously at multiple sites. Each rabbit was immunized twice at a 2-week interval with approximately 1 mg/kg of purified recombinant proteins in an equal volume of Freund complete adjuvant. Then, all the serum samples were obtained when the second booster immunization was administered 10 days later.

### Recombinant proteins binding assays by far-western blot

Binding studies were performed by Far-Western blotting. Recombinant proteins were separated by SDS-PAGE, then electroblotted onto PVDF membrane and blocked with 5% (w/v) skimmed milk at 4°C overnight. Subsequently, the membrane was incubated with human LN (Sigma; 5 μg/ml) or human FN (Sigma; 10 μg/ml) for 24 h at 4°C. Frequently, the membranes were washed three times with TBST and incubated with rabbit anti-LN (Abcam; 1:2000 dilution) or rabbit anti-FN (Boster; 1:2000 dilution) antibody at 37°C for 2 h, rinsed three times with TBST and then incubated with goat anti-rabbit IgG antibody (Boster; 1:2000 dilution). The specific bands were developed using substrate solution 3,3′-diaminobenzidine (DAB; Tiangen, China). The recombinant protein MRP was performed as negative control.

### Recombinant proteins binding assays by ELISA

Quantitative analysis of the binding efficiency was carried out following the previous report with some modifications (Esgleas et al., 2008). Briefly, Microtitre 96-well plates were coated overnight at 4°C with 100 μl of 5 μg/ml purified recombinant proteins. Casein coated wells were used as negative controls for non-specific adherence of LN or FN. The wells were rinsed three times with PBST [PBS, pH 7.4, containing 0.05% (v/v) Tween 20]. Then 200 μl 0.5% bovine serum albumin (BSA; Sigma) in PBST was added. Following the incubation, the plates were washed three times with PBST. Thereafter, 100 μl serially diluted of LN or FN was added and the wells were incubated at 37°C for 2 h. After several washes with PBST, rabbit anti-LN (Abcam; 1:2000 dilution) or rabbit anti-FN (Boster; 1:2000 dilution) antibody were added to every plate. The wells were incubated at 37°C for another 2 h, followed by three washes with PBST. The secondary antibody goat anti-rabbit IgG (Boster; 1:5000 dilution) were added to the corresponding plates and wells were then incubated at 37°C for 1 h. 3,3′,5,5′-tetramethylbenzidine served as the enzyme substrate according to the manufacturer's instructions. The chromogenic reaction was stopped by adding 50 μl of H_2_SO_4_ (2 M) to each plate and the wells were read at 450 nm with a microplate reader.

### Indirect immunofluorescence assays

Immunofluorescence assays were conducted to visualize whether recombinant proteins can specifically bind to the Hep-2 cells. The cells in 24-well cell plates were performed as the customary procedure: washed two times with PBS, fixed for 20 min with cold acetone at −20°C, and then air-dried. The fixed Hep-2 cells (approximately 6 × 10^5^ cells/well) were incubated with 100 μg/ml of purified recombinant proteins or BSA for 1 h at 37°C, and then washed three more times with PBS. His tag monoclonal antibody (Boster; 1:1000 dilution) were added to every plate and wells were then incubated at 37°C for 30 min. After washing, the cultured cells were incubated with goat anti-mouse IgG-FITC (Boster; 1:2000 dilution) at 37°C for 30 min. After a finial wash, the samples were examined using a fluorescence microscope (ZEISS, Germany).

### Competitive inhibition assays

To evaluate the contribution of purified recombinant proteins to the adhesion of SS2, the inhibitions of the purified proteins and their corresponding polyclonal antibodies were determined. The adherence assay was performed to determine the ability of recombinant proteins to bind to the surface of Hep-2 cells. Hep-2 cells were cultured in 24-well tissue culture plates (approximately 6 × 10^5^ cells/well), and then washed three times with PBS before treated with 100 μg/ml of purified recombinant proteins at 37°C for 2 h. Additionally, cells incubated with 100 μg/ml of BSA were served as control. All cells were rinsed three times with PBS, and infected with the diluted SS2 at MOI 1:20 (cell: bacterium) in 1 ml Dulbecco's Modified Eagle's Medium (DMEM) at concentration of 1.2 × 10^7^ CFU/ml. Then, the wells were centrifuged for 10 min at 800 × g and incubated at 4°C for 2 h. Following the incubation, the wells were washed with PBS seven times to remove unbound SS2 and then treated with a lysis buffer containing 0.1% trypsin and 0.025% (v/v) Triton X-100, followed by agar plating and bacterial enumeration.

The inhibitory assays of their polyclonal antibodies were also assessed. SS2 (1.2 × 10^7^ CFU/ml) were pre-incubated with the polyclonal antibodies (1:20 dilution) that against recombinant proteins at 37°C for 30 min. SS2 which were pre-incubated with preimmune sera (1:20 dilution) were served as a control. The bacterial suspension dissolved in DMEM was added in every well, and then the wells were centrifuged for 10 min at 800 × g and incubated at 4°C for 2 h. Following the incubation, the wells were washed with PBS seven times to remove unbound SS2 and then treated with a lysis buffer containing 0.1% trypsin and 0.025% (v/v) Triton X-100, followed by agar plating and bacterial enumeration. All experiments were performed in triplicate wells and repeated 3 times.

### Statistical analysis

All the assays were repeated at least three times, and the data were analyzed using Student's *t*-test with SPSS 15.0. For all tests, a value of *P* < 0.05 was considered as the threshold for significance.

## Results

### Identification of novel SS2 LN- and FN-binding proteins by proteomic and far-western blotting assays

**Figure 1:**
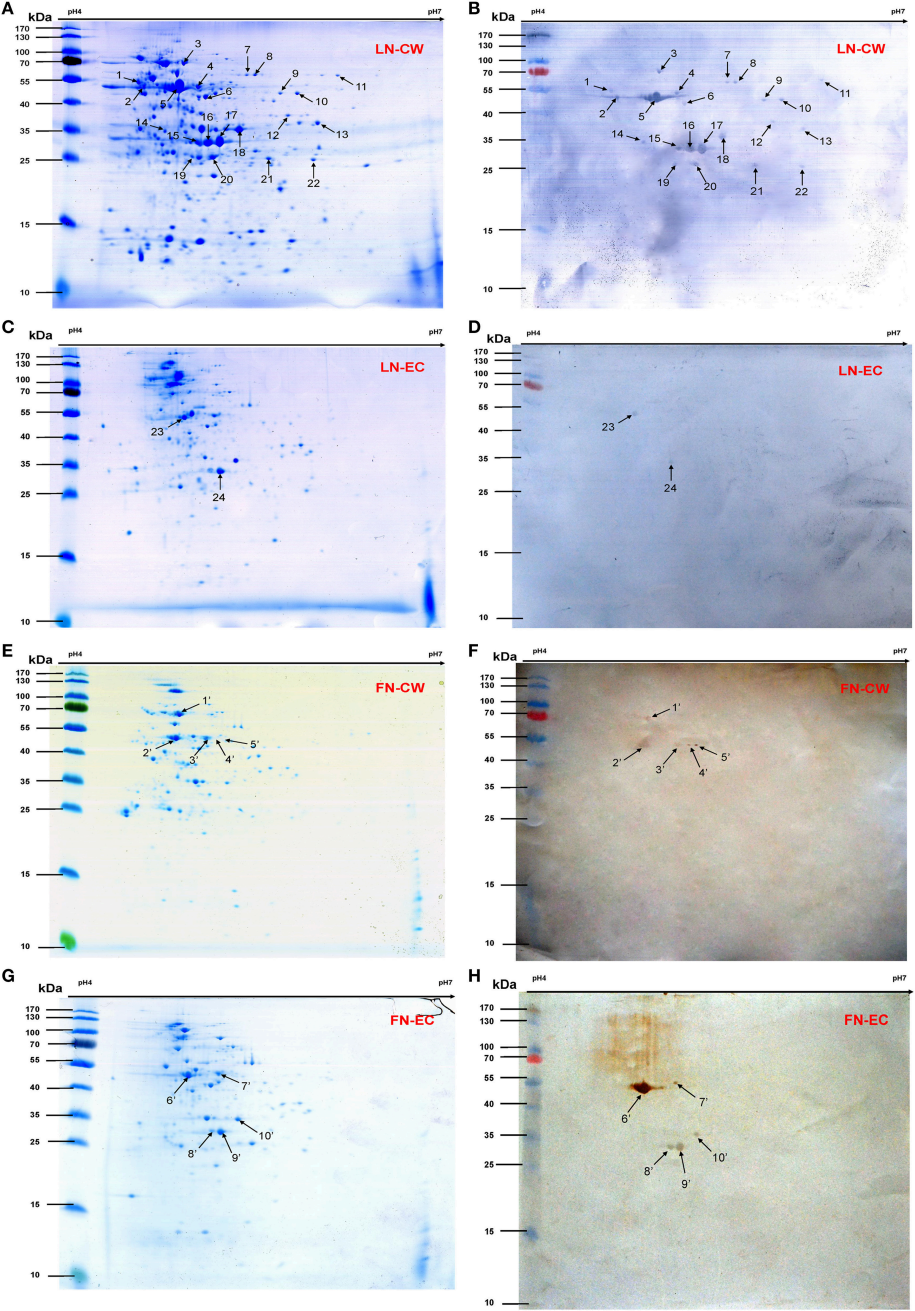
**2-DE gels and Far-Western blot identification of LN- and FN-binding proteins of SS2 CW and EC**. The CW and EC proteins were separated in the first dimension by IEF in the pI range of 4–7 and by 12% SDS-PAGE in the second dimension, and then the 2-DE gels transferred to PVDF and incubated with LN or LN. Arrows indicate potential LN- or FN-binding proteins recognized with goat anti-rabbit IgG antibody. **(A)** 2-DE gel of SS2 CW proteins. **(B)** Far-Western blot of CW proteins incubated with LN (LN-CW). **(C)** 2-DE gel of SS2 EC proteins. **(D)** Far-Western blot of EC proteins incubated with LN (LN-EC). **(E)** 2-DE gel of SS2 CW proteins. **(F)** Far-Western blot of CW proteins incubated with FN (FN-CW). **(G)** 2-DE gel of SS2 EC proteins. **(H)** Far-Western blot of EC proteins incubated with FN (FN-EC).

Bacterial CW and EC proteins from SS2 were separated by 2D SDS-PAGE over a pH range of 4–7 and transferred to PVDF membranes. Consistent with our previously study, at least 200 Coomassie brilliant blue G250 stained spots were identified on the 2-DE gels (Figures 1A,C). After incubation with LN, 22 LN reactive spots were identified in CW and two in EC (Figures 1B,D). By using the same approach, SS2 CW and EC protein spots in the 2-DE gels were stained with the Coomassie brilliant blue G250 (Figures 1E,G) and five potential SS2 proteins in CW and five in EC that bound to FN were observed (Figures 1F,H). No specific protein spots were observed in the negative control. All the spots were then excised and identified by MALDI-TOF-MS and data were searched against the NCBI sequence database for match. Taken together, proteomic analysis together with Far-Western blotting assays identified 15 potential LN-binding proteins and five potential FN-binding proteins from SS2 surface proteins. All the 15 potential LN-binding proteins and two potential FN-binding proteins were detected for the first time. Five proteins (OppA, EF-Tu, enolase, LDH, and FBA) showed specific binding affinities for both LN and FN. The probability score, number of peptide matches, isoelectric point (p*I*) and molecular weight (MW) enabled confirmed spot identification. All the results are summarized in Table 2.

**Table 2:** **LN- and FN-binding proteins identified by MALDI-TOF-MS and bioinformatics analysis**.

**Spot no.^a^**	**Identified protein**	**Accession no**.	**Theoretical *p*I/MW^b^**	**Experimental *p*I/MW**	**Mascot score**	**Coverage (%)**	**PSORT prediction^c^**	**Reported surface protein**
1	Amino acid ABC transporter permease	WP_012775143.1	4.55/53192	4.3/53000	105	25	Unknown	
2, 5, 23	enolase	YP_001201070.1	4.66/47066	4.7/50000	340	69	Cytoplasmic	Yes
3	Oligopeptide-binding protein OppA precursor	YP_003025672.1	4.89/65672	4.8/66000	211	49	Unknown	
4	Elongation factor Tu	YP_001197896.1	4.87/44755	4.9/47000	164	40	Cytoplasmic	
6	Phosphoglycerate kinase	WP_029998367.1	4.86/42053	5.0/42000	208	62	Cytoplasmic	
7,8	Pyruvate kinase	WP_029186723.1	5.24/54836	5.3/56000	290	58	Cytoplasmic	
9,10	Glutamate dehydrogenase	ACG49984.1	5.43/48811	5.6/46000	162	37	Cytoplasmic	Yes
11	Inosine 5′-monophosphate dehydrogenase	WP_023371888.1	5.69/52748	6.1/56000	244	45	Cytoplasmic	
12	Glyceraldehyde-3-phosphate dehydrogenase	AAZ78247.1	5.58/35883	5.6/37000	130	40	Cytoplasmic	Yes
13	6-phosphofructokinase	WP_029188183.1	5.32/35360	5.9/36000	191	55	Cytoplasmic	
14	Pyrophosphatase	WP_009910564.1	4.59/33367	4.5/35000	321	54	Cytoplasmic	
15, 16, 17, 20	Fructose-bisphosphate aldolase	WP_029185467.1	4.90/31157	5.0/31000	134	49	Cytoplasmic	
18, 19	Lactate dehydrogenase	WP_029999076.1	5.14/35390	5.2/36000	276	48	Cytoplasmic	
21, 22	3-ketoacyl-ACP reductase	WP_032511883.1	5.29/25533	5.5/25000	213	84	Cytoplasmic	
24	Cell wall surface anchor protein	WP_012027978.1	5.75/32143	5.1/31000	107	36	Unknown	
1′	Oligopeptide-binding protein OppA precursor	YP_003025672.1	4.89/65672	4.8/66000	253	35	Unknown	
2′, 6′	enolase	YP_001201070.1	4.66/47066	4.7/50000	256	37	Cytoplasmic	Yes
3′, 4′, 5′, 7′	Elongation factor Tu	YP_001197896.1	4.87/44755	4.9/47000	228	46	Cytoplasmic	
8′, 9′	Fructose-bisphosphate aldolase	WP_029185467.1	4.90/31157	5.0/31000	178	42	Cytoplasmic	
10′	Lactate dehydrogenase	WP_029999076.1	5.14/35390	5.2/36000	276	48	Cytoplasmic	

a *Spot numbers correspond to those indicated in Figure 1*.

b *Theoretical pI and MW were calculated by Compute pI/Mw server (http://web.expasy.org/compute_pi/)*.

c *Subcellular location of identified proteins is predicted by PSORTb (http://www.psort.org/psortb/index.html)*.

### Recombinant proteins have LN- and FN-binding activities

**Figure 2:**
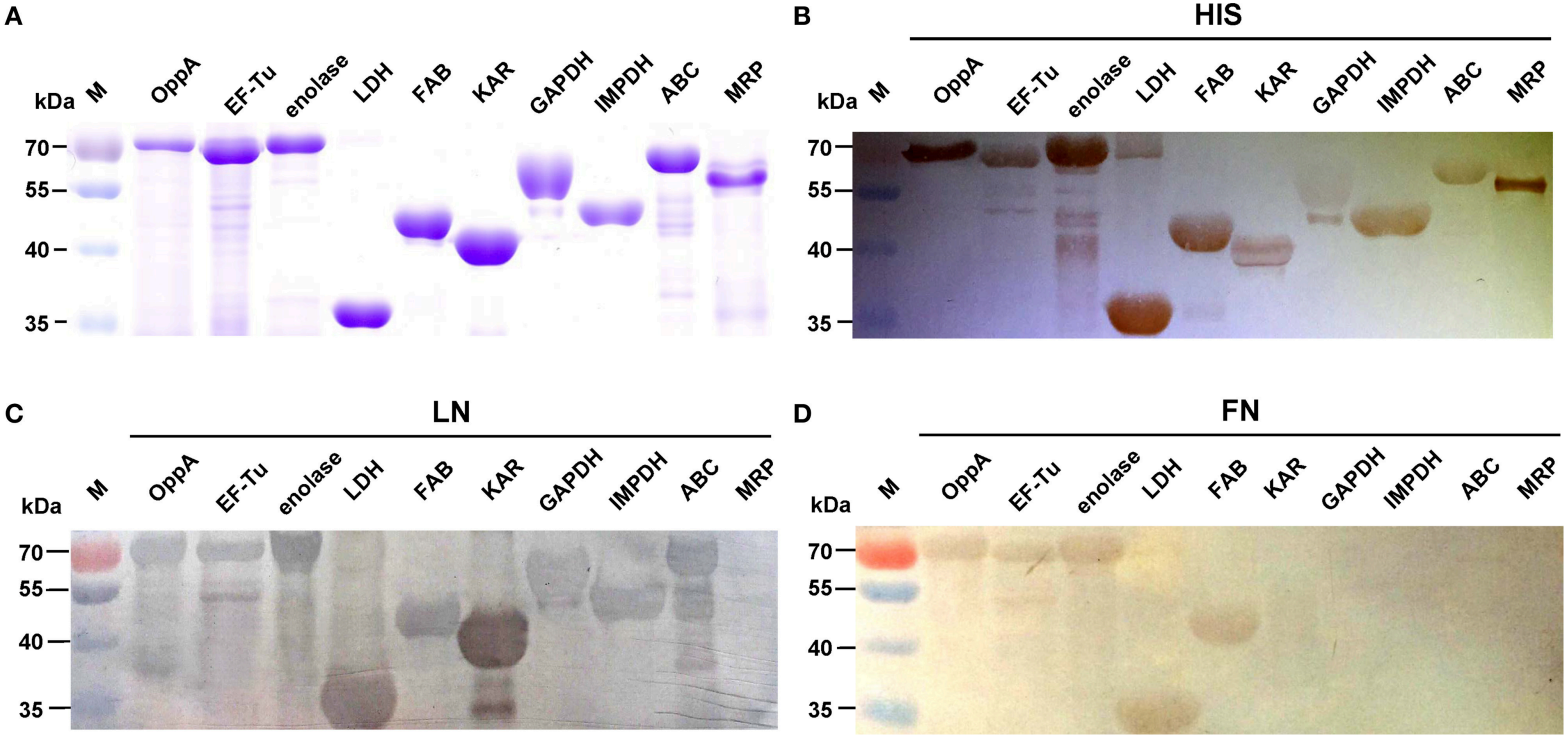
**Determine the binding of the recombinant proteins to LN and FN by Far-Western blot**. Coomassie G-250-stained gel **(A)**, Western blot analysis **(B)**, and Far-Western blot analysis **(C,D)** of the SS2 recombinant proteins. Recombinant proteins were separated by 12% SDS-PAGE, then transferred to PVDF membrane and incubated with human LN or LN. Bound LN or FN was detected with goat anti-rabbit IgG antibody. The Western blot **(B)** was probed with his tag monoclonal antibody (Boster).

**Figure 3:**
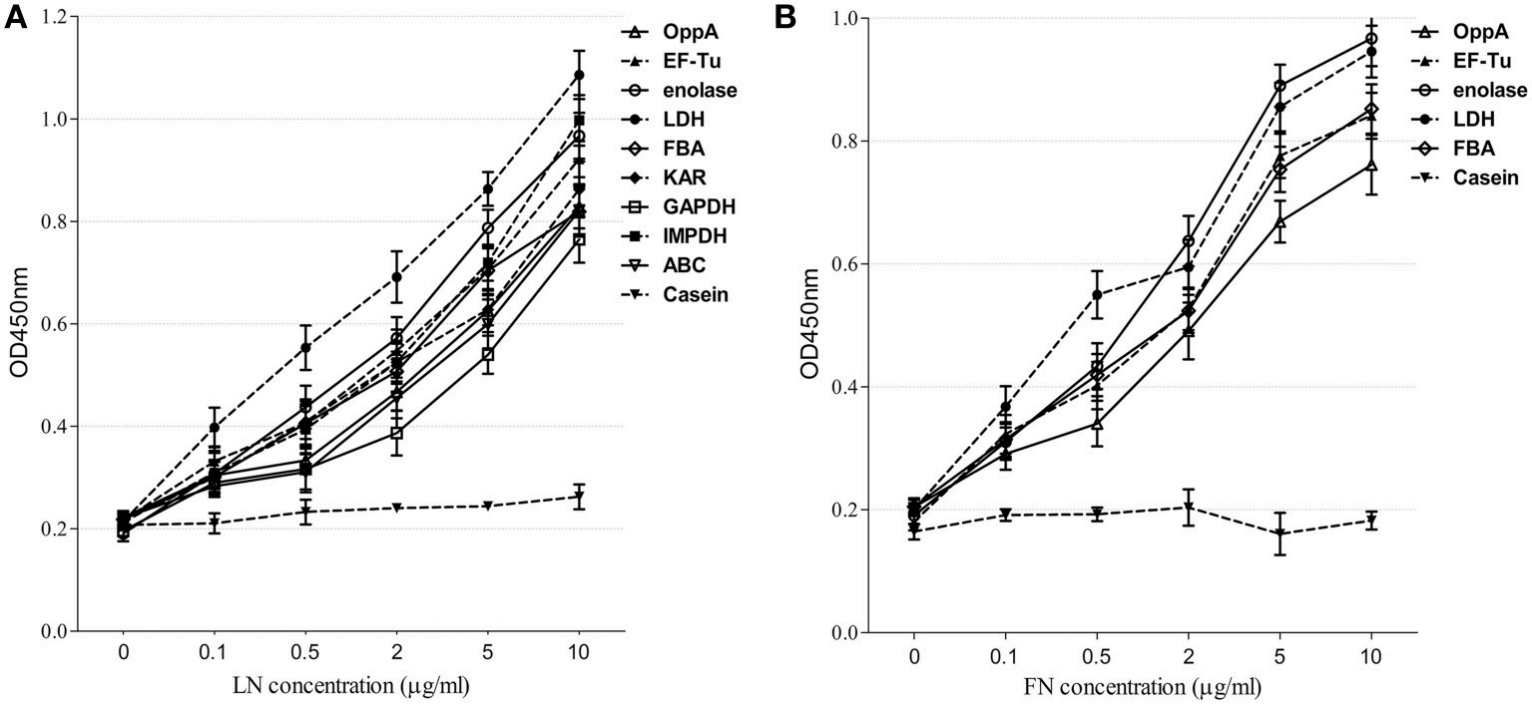
**Determine the binding of the recombinant proteins to LN and FN by ELISA**. Microtiter plates were coated with 100 μl of 5 μg/ml purified recombinant proteins and reacted with the indicated concentrations of human LN **(A)** or FN **(B)**. The negative control was coated with 100 μl of 5 μg/ml casein and incubated with the indicated concentrations of human LN or FN. Binding was evaluated after 2 h. Bound LN or FN was detected with goat anti-rabbit IgG antibody. Data are expressed as the mean ± SD of three independent experiments performed in triplicate.

We selected nine potential LN- or FN-binding proteins for expression and further confirmed by Far-Western blotting and ELISA. After purification by Ni-chelating chromatography, the SDS-PAGE (Figure 2A) and Western blotting analysis (Figure 2B) of the recombinant proteins revealed that the His-tagged fusion proteins were successfully purified. The LN-binding properties of purified recombinant OppA, EF-Tu, enolase, LDH, FBA, KAR, GAPDH, IMPDH, and ABC were verified by Far-Western blot analysis (Figure 2C). A specific response to human FN was also detected for purified recombinant OppA, EF-Tu, enolase, LDH, FBA on the PVDF membrane (Figure 2D). Notably, LN and FN failed to interact with the negative control protein MRP. Qualitatively, the ability of purified recombinant proteins to bind LN or FN was further evaluated by ELISA. The result indicates that the recombinant captured proteins in SS2 interact specifically with LN (Figure 3A) and FN (Figure 3B), and purified recombinant proteins showed concentration dependent binding to human LN or FN. Under similar assay conditions, LN and FN failed to interact with casein, which was served as a negative control. These results provide interesting insights into the role of LN or FN-binding surface proteins of SS2 and clues to the pathogenesis of the SS2 infection.

### Confirmation of the interaction of Hep-2 cells with the identified LN- or FN-binding proteins

**Figure 4:**
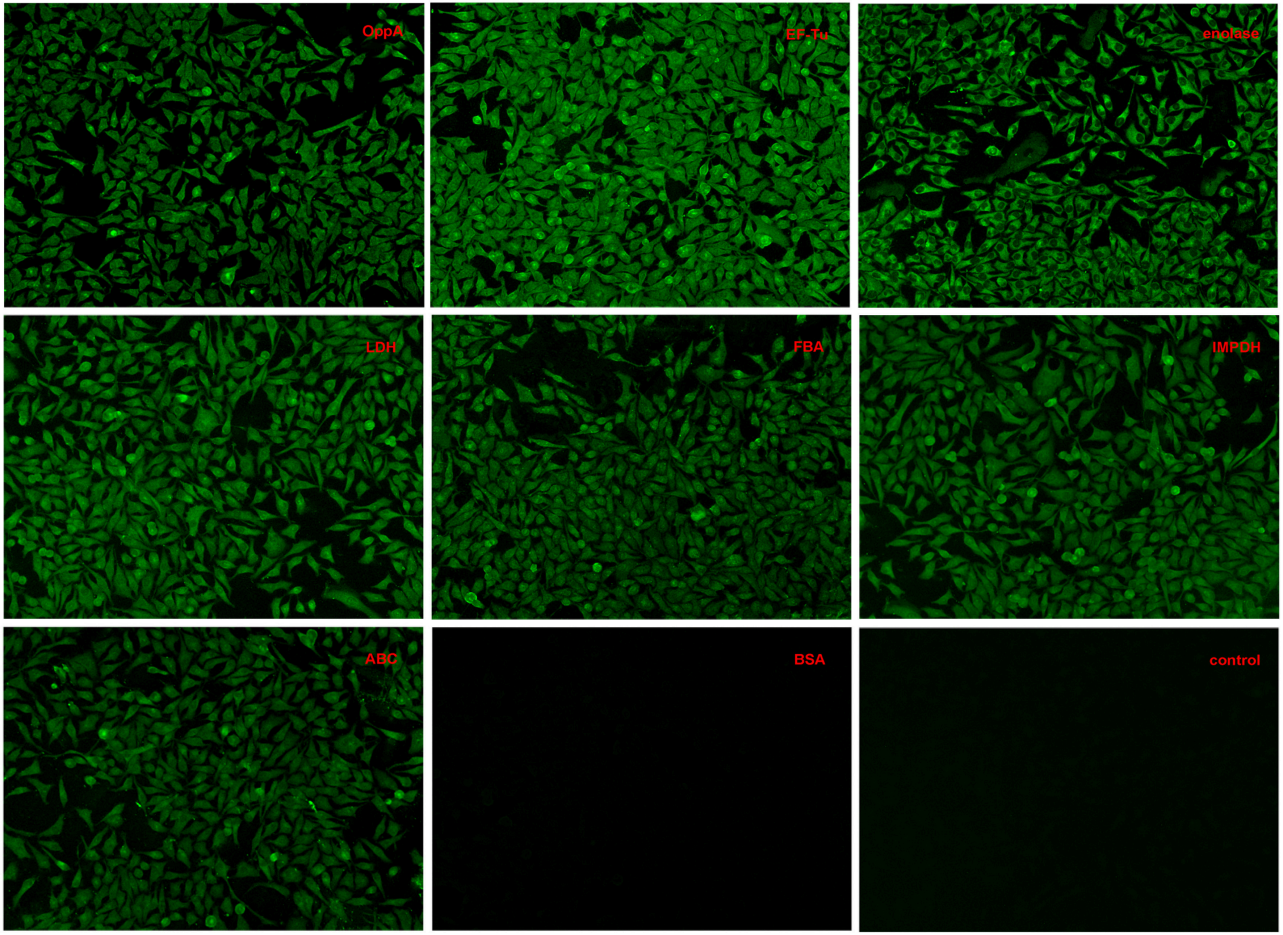
**Adherence of recombinant proteins to Hep-2 cells confirmed by an indirect immunofluorescence assay**. Hep-2 cells were incubated with purified recombinant OppA, EF-Tu, enolase, LDH, FBA, IMPDH, ABC, or BSA, and then incubated with rabbit antibodies against the corresponding recombinant proteins, stained with goat anti-rabbit IgG-FITC before examined with a fluorescence microscope. The secondary antibody goat anti-rabbit IgG-FITC was used alone to stain Hep-2 cells as a blank control.

Indirect immunofluorescence analyses were conducted to test whether recombinant OppA, EF-Tu, enolase, LDH, FBA, IMPDH, and ABC contributed to SS2 adhere to host cells. Among these proteins, the binding activity of SS2 enolase and GAPDH to Hep-2 cells has been proven in the previous study (Wang and Lu, 2007; Zhang et al., 2009a). It has been reported that enolase binds specifically to fibrinogen (Esgleas et al., 2008; Pian et al., 2015), collagen (Esgleas et al., 2008; Zhang et al., 2014a; Pian et al., 2015), fibronectin, and plasminogen (Esgleas et al., 2008; Pian et al., 2015), but its LN-binding property was identified for the first time in this study. The further analysis of enolase may give some insights in the pathogenesis of SS infection. So the protein enolase was used as an internal control and GAPDH was not further studied. Similar to enolase, obvious green signal was observed from the surface of Hep-2 cells pre-incubated with the seven purified recombinant proteins, while no significant green signal was detected from the negative control (BSA) and the blank control (the secondary antibody was used alone to stain Hep-2 cells, Figure 4). The results demonstrate that all seven purified recombinant proteins could interact with Hep-2 cells. Furthermore, we conclude that ECM-binding proteins may contribute to SS2 binding to host cells.

### Identification of EF-Tu, enolase, LDH, and FBA as four major SS2 adhesins

**Figure 5:**
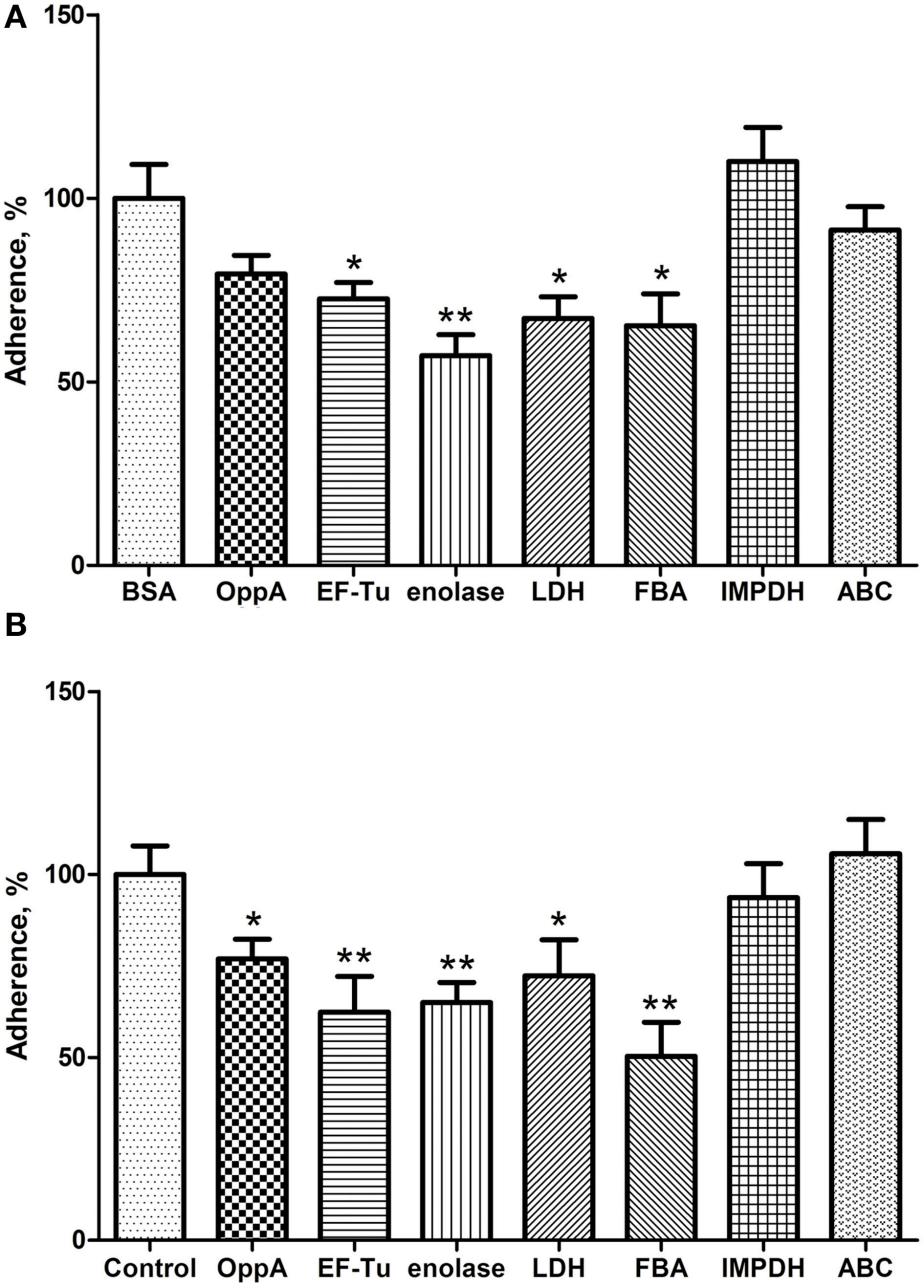
**EF-Tu, enolase, LDH, and FBA are four major SS2 adhesins confirmed by competition inhibition assays**. Inhibition of SS2 adhesion to Hep-2 cells by the recombinant proteins **(A)** and their corresponding polyclonal antibodies **(B)**. Data are expressed as the mean ± SD of three independent experiments. Significant differences are indicated (^**^P < 0.01; ^*^P < 0.05).

To assess the contribution of recombinant proteins to the adhesion of SS2, two inhibition assays were performed at the same time. In the protein inhibition assay, Hep-2 cells were treated with the purified OppA, EF-Tu, enolase, LDH, FBA, IMPDH, and ABC before SS2 adhering to the Hep-2 cells, and BSA was used as negative control. Compared with BSA, recombinant EF-Tu, enolase, LDH, and FBA were found to decrease the adhesion to Hep-2 cells by 27.3, 42.9, 32.7, and 34.7% respectively, and no significant inhibition with other recombinant proteins (Figure 5A). In the antibodies inhibition assay, the polyclonal antibodies against recombinant OppA, EF-Tu, enolase, LDH, and FBA were able to decrease SS2 adherence to Hep-2 cells compared to pre-immune sera (Figure 5B). The level of adherence is expressed as the percent of adherence of ZY05719 without antibody. The results showed that EF-Tu, enolase, LDH, and FBA contribute to the adherence of SS2 to host cells. We consider these proteins to be four major SS2 adhesins.

## Discussion

SS is a major swine pathogen that interacts with components of the host ECM, such as LN and FN, to initiate colonization (Esgleas et al., 2005). It has been demonstrated that the pathogens can invade host epithelial cells through their own ECM-binding proteins (Molinari et al., 1997; Henderson et al., 2011). The interactions between host ECM proteins and bacterial surface adhesins is a necessary step in the colonization of epithelial surfaces, and has also been implicated in bacterial invasion of host cells (Tamura et al., 1994). Fortunately, the CW and EC proteins of SS2 are suitable for identification by 2D-Far-Western blot because most of the SS2 surface proteins are soluble in nature (Jungblut, 2001; Zhang and Lu, 2007). Therefore, the purpose of this study was to identify proteins with LN- and FN-binding activity from SS2 by a useful approach and to give insight into the pathogenesis of the SS2 infection.

The identification of novel SS2 surface proteins binding to human LN and human FN was developed combining proteomic analysis and Far-Western blotting assays. By using this approach, 15 novel LN-binding proteins and two novel FN-binding proteins were identified from SS2 surface proteins. Nine proteins were selected from the identified prey for further investigation by Far-Western blotting and ELISA. The results indicate that all the selected recombinant proteins interact specifically with LN or FN. Furthermore, five of them exhibited specifically binding activity to both human LN and human FN. Under similar assay conditions, LN and FN failed to interact with the negative control proteins, casein and MRP. Therefore, evidences strongly demonstrate that the approach described here represents an accurate tool for investigating the ECM-binding proteins.

In this work, most of the identified proteins were predicted to be cytoplasmic by using PSORTb software (http://www.psort.org/psortb/index.html), but many reportedly cytoplasmic proteins are located on the bacterial surface, actually. So far, four of the SS2 cytoplasmic proteins (FBPS, GAPDH, GDH, and enolase) have been reported as surface proteins in previous studies (Okwumabua et al., 2001; de Greeff et al., 2002; Yu et al., 2005; Wang and Lu, 2007; Zhang et al., 2009a) and suggested to participate in adhesion to host cells (Fittipaldi et al., 2012). Therefore, the roles of the cytoplasmic proteins in the interactions between bacteria and the host should not be ignored. The mechanism by which these cytoplasmic proteins (non-LPXTG proteins) are translocated to the cell surface is still unknown. Notably, non-LPXTG proteins have been reported can play an important role in the adhesion of SS2 to ECM proteins (Vanier et al., 2008). Some studies demonstrate that LPXTG-containing surface proteins play a major role in the adhesion to the components of ECM, but we think the interactions of SS2 proteins with host cells are not directly related to the presence of an LPXTG motif. On the basis of these results, we consider the proteins captured in this study can participate in the interactions between the ECM and SS2. As is shown in Figure 4, all of the recombinant proteins could adhere to the Hep-2 cells. We also examined a quantitative analysis of these proteins to SS2 adherence to Hep-2 cells by competitive inhibition assays. The results indicate that recombinant EF-Tu, enolase, LDH, and FBA play a critical role in the bacterial adherence process. One of the recombinant protein, enolase, was used as a positive control in this work because of the adhesion and the inhibition have been reported in the previous study (Feng et al., 2009; Zhang et al., 2009a; Chen et al., 2011). Enolase is localized at the surface of SS2 and has been reported to bind fibrinogen (Pian et al., 2015), collagen (Zhang et al., 2014a), fibronectin, and plasminogen (Esgleas et al., 2008). To date, there is no report of its LN-binding ability. To our knowledge, OppA and FBA have been reported as FN-binding proteins, however, neither the LN-binding activity of OppA, EF-Tu, LDH, FBA, IMPDH, and ABC nor the critical roles of them in the adhesion of SS2 to host cells have been reported before.

Although, the roles of many captured proteins are uncertain, the potential roles of these proteins in host-SS2 interactions cannot be ignored. Phosphoglycerate kinase (PGK) is an immunogenic protein from SS biofilms and have FN-binding activity (Wang et al., 2012; Zhang et al., 2014a). Glutamate dehydrogenase (GDH) is a cell surface protein that serves as an antigen for the detection of SS infection (Okwumabua et al., 2001). Inosine 5′-monophosphate dehydrogenase (IMPDH) was shown to have collagen-binding activity in previous study (Zhang et al., 2014a), and considered to be a potential virulence factor of SS (Zhang et al., 2009b). Glyceraldehyde-3-phosphate dehydrogenase (GAPDH) has been reported in bacterial adherence and is able to clearly decrease the adherence of SS to Hep-2 cells and porcine tracheal rings (Brassard et al., 2004; Wang and Lu, 2007). Fructose-bisphosphate aldolase (FBA) has been identified as an immunogenic surface protein of *S. suis* serotype 9 (Wu et al., 2008). This cell surface associated protein affords protective immune responses and elicits significant protection against respiratory challenge with virulent *S. pneumoniae* (Ling et al., 2004). The identified proteins can serve as a foundation for further research of the roles that they play in the pathogenesis or virulence of SS2.

The major finding of the present study was the identification of 15 novel LN-binding proteins and two novel FN-binding proteins in SS2 surface proteins. Recombinant proteins, such as OppA, EF-Tu, and enolase, LDH, FBA, KAR, GAPDH, IMPDH, and ABC, which have LN- or FN-binding activities, were further confirmed by Far-Western blotting and ELISA. Adherence assays indicate that ECM-binding proteins may contribute to SS2 binding to host cells. Further, analyses revealed that EF-Tu, enolase, LDH, and FBA, are major adhesins that participate in the adhesion of SS2 to Hep-2 cells. Moreover, this study also reveals many new potential adhesins and provides promising candidates for therapies in vaccination strategies.

### Conflict of interest statement

The authors declare that the research was conducted in the absence of any commercial or financial relationships that could be construed as a potential conflict of interest.
