# Corrigendum: Identification of Novel Laminin- and Fibronectin-Binding Proteins by Far-Western Blot: Capturing the Adhesins of *Streptococcus suis* Type 2

**DOI:** 10.3389/fcimb.2020.593413

**Published:** 2020-10-29

**Authors:** Quan Li, Hanze Liu, Dechao Du, Yanfei Yu, Caifeng Ma, Fangfang Jiao, Huochun Yao, Chengping Lu, Wei Zhang

**Affiliations:** Key Lab of Animal Bacteriology, Ministry of Agriculture, College of Veterinary Medicine, China OIE Reference Lab for Swine Streptococcosis, Nanjing Agricultural University, Nanjing, China

**Keywords:** *Streptococcus suis* serotype 2, interactions, surface proteins, LN- binding proteins, FN-binding proteins

In the original article, there was a mistake in the legend for **Figures 1** and **2** as published. There are some creases and non-specific stains appear in the original **Figures 1F, H** and **2C**. In order to improve the presentation quality of the figures, we modified the creases and non-specific stains outside of the results. These modifications do not affect the results of the article. This clarification needs to be added to the figure legends of these figures. The correct legends appears below.

**Figure 1:**
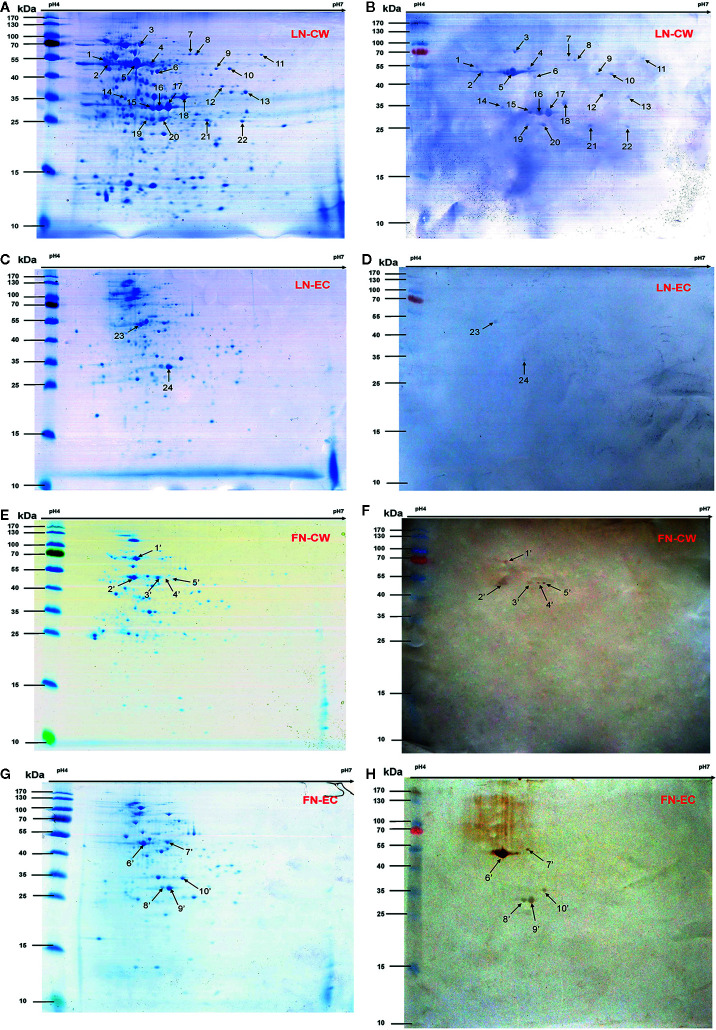
2-DE gels and Far-Western blot identification of LN- and FN-binding proteins of SS2 CW and EC. The CW and EC proteins were separated in the first dimension by IEF in the pI range of 4–7 and by 12% SDS-PAGE in the second dimension, and then the 2-DE gels transferred to PVDF and incubated with LN or LN. Arrows indicate potential LN- or FN-binding proteins recognized with goat anti-rabbit IgG antibody. **(A)** 2-DE gel of SS2 CW proteins. **(B)** Far-Western blot of CW proteins incubated with LN (LN-CW). **(C)** 2-DE gel of SS2 EC proteins. **(D)** Far-Western blot of EC proteins incubated with LN (LN-EC). **(E)** 2-DE gel of SS2 CW proteins. **(F)** Far-Western blot of CW proteins incubated with FN (FN-CW). **(G)** 2-DE gel of SS2 EC proteins. **(H)** Far-Western blot of EC proteins incubated with FN (FN-EC). In order to improve the presentation quality of **(F, H)**, we modified the creases and non-specific stains outside of the results. These modifications do not affect the results of the article.

**Figure 2:**
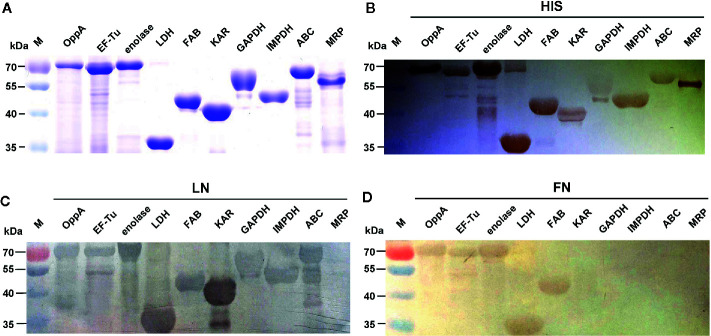
Determine the binding of the recombinant proteins to LN and FN by Far-Western blot. Coomassie G-250-stained gel **(A)**, Western blot analysis **(B)**, and Far-Western blot analysis **(C, D)** of the SS2 recombinant proteins. Recombinant proteins were separated by 12% SDS-PAGE, then transferred to PVDF membrane and incubated with human LN or LN. Bound LN or FN was detected with goat anti-rabbit IgG antibody. The Western blot **(B)** was probed with his tag monoclonal antibody (Boster). In order to improve the presentation quality of **(C)**, we modified the creases outside of the results. These modifications do not affect the results of the article.

In the original article, there was a mistake in **Figure 4** as published. Each sample hole was photographed twice or more for the same or different field of view. Due to the similarity in the fluorescence intensity of LDH protein and IMPDH protein, we made a mistake while selecting the pictures among a lot of pictures. Another LDH picture from the same hole with different views was incorrectly named IMPDH. The corrected **Figure 4** appears below.

**Figure 4:**
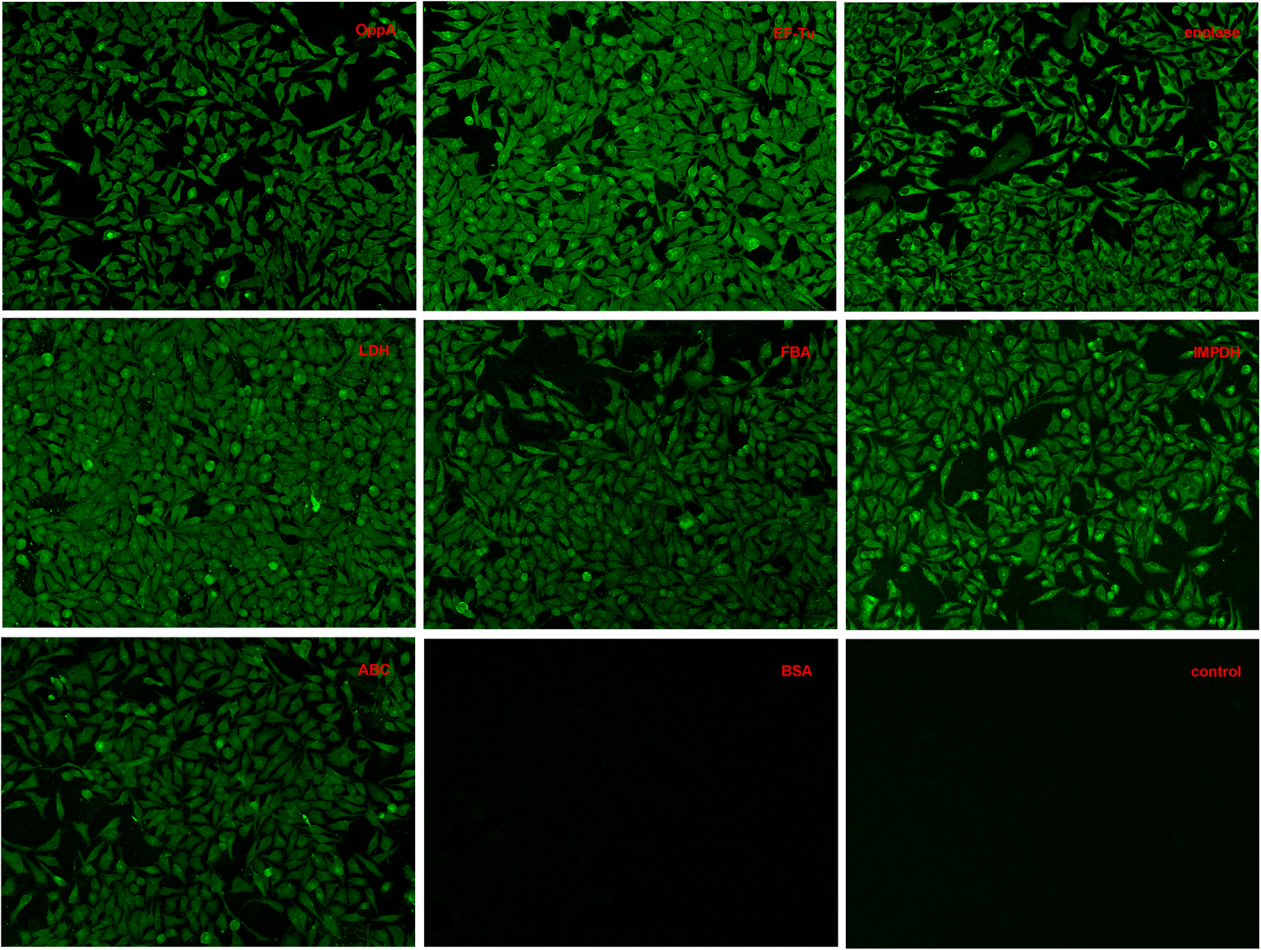
Adherence of recombinant proteins to Hep-2 cells confirmed by an indirect immunofluorescence assay.

The authors apologize for these errors and state that this does not change the scientific conclusions of the article in any way. The original article has been updated.

